# Factors associated with knowledge, attitudes, and practices of mixed crop-livestock farmers on Crimean-Congo hemorrhagic fever (CCHF) and other zoonoses in Burkina Faso

**DOI:** 10.1016/j.onehlt.2025.101066

**Published:** 2025-05-08

**Authors:** Abdoul Kader Ilboudo, Michel Dione, Ard M. Nijhof, Martin H. Groschup, Ousmane Traoré, Guy S. Ilboudo, Zekiba Tarnagda, Madi Savadogo, Bernard Bett

**Affiliations:** a International Livestock Research Institute (ILRI), Human and Animal Health, Kenya; b Freie Universität Berlin, Institute for Parasitology and Tropical Veterinary Medicine, Berlin, Germany; c Freie Universität Berlin, Veterinary Centre for Resistance Research, Berlin, Germany; d Institut de Recherche en Sciences de la Santé, National Influenza Reference Laboratory, Unit of Epidemic Prone Diseases, Emerging Diseases and Zoonoses, Department of Medical Biology and Public Health (IRSS/CNRST), Ouagadougou, Burkina Faso; e Directorate of Animal Health, Directorate General of Veterinary Services, Ministry of Agriculture, Animal et Halieutic Resources, Ouagadougou, Burkina Faso; f Université Norbert Zongo, Economics and Management Sciences Research Unit, Koudougou, Burkina Faso; g Friedrich-Loeffler -Institut, Institute of Novel and Emerging Diseases, Insel Riems, Germany; h International Livestock Research Institute (ILRI), Human and Animal Health, Burkina Faso

**Keywords:** Knowledge, Attitudes, Practices, Crimean Congo hemorrhagic fever, Seemingly unrelated regression, Zoonoses, Burkina Faso

## Abstract

**Background:**

The burden of zoonotic diseases remains high in low and middle-income countries. Among the most prevalent zoonoses, Crimean-Congo hemorrhagic fever (CCHF) can pose economic and health threats, particularly among at-risk professionals. We aimed to assess the knowledge, attitudes, and practices regarding CCHF and other zoonoses among mixed-crop livestock farmers in the rural settings of Burkina Faso.

**Methods:**

A cross-sectional study that involved selected households from sixteen villages was conducted. Consenting participants aged six and above were randomly included, and a structured questionnaire that collected socio-economic data, knowledge, attitudes, and practices concerning CCHF and other zoonoses was administered. Two index outcome variables were created based on an elaborated scale: i) attitudes and practices at risk of CCHF; and ii) knowledge of zoonoses. Descriptive statistics were performed, and univariable ordinary least squares (OLS) and seemingly unrelated regression (SUR) were used for univariable and multivariable modeling, respectively, to assess the drivers of both outcomes.

**Results:**

Of the 717 respondents, 66.4 % were male, and 20.4 % were under 15 years old. The attitudes and practices at risk were high (48.1 %), and the knowledge toward zoonoses was limited for 47.8 % of the farmers. Our multivariable SUR model shows higher odds of attitudes and practices associated with CCHF risk in men (Coef [95 %CI] = 2.85[2.14;3.56]; *p*-value<0.001). This risk increases with the distance to the livestock grazing area, and among the households owning their livestock grazing area (Coef [95 %CI] = 1.57[0.47;2.66]; *p*-value = 0.005). The farmers' age (Coef[95 % CI] = 0.02[0.002;0.04]; *p*-value = 0.028), the male gender (Coef 95 %CI] = 1.5[0.94;2.14]; p-value<0.001), the household's farming surface (Coef[95 %CI] = 0.03[0.002;0.6]; p-value = 0.032), were the significant factors driving knowledge of zoonoses among the farmers.

**Conclusion:**

The study reveals concerning high-risk behavior associated with CCHF among mixed-crop livestock farmers in rural Burkina Faso. The identified socio-demographic drivers underscore the importance of targeted educational and preventive measures to mitigate the impact of CCHF in this vulnerable population.

## 1 Introduction

Zoonoses are diseases or infections that can be naturally transmitted from vertebrate animals to humans and vice versa [1]. They are the most frequently reported emerging infectious diseases (EIDs) and present significant public health challenges globally [2]. Particularly prevalent in the Sub-Saharan Africa (SSA) region, where close cohabitation of humans, domestic animals, and wildlife in rural communities facilitates disease transmission [3], zoonoses not only impact human health but also threaten livestock productivity and food security [4]. Among the most common zoonotic infections in SSA are Rift Valley fever, Lassa fever, brucellosis, rabies, avian influenza (H5N1), and Crimean-Congo hemorrhagic fever (CCHF) [5].

CCHF is a zoonotic tick-borne viral infection that occurs in many countries in Eastern Europe, Africa, Asia, and the Middle East. Hundreds of outbreaks have occurred worldwide, with up to 20,000 confirmed cases and case-fatality rates ranging from 5 to 80 % [6,7]. CCHFV is transmitted to humans via tick bites or directly through contact with an infected patient during the highly viremic phase or by handling the blood or flesh of an infected animal [7,8]. In SSA, human case of CCHF was first discovered in the Democratic Republic of Congo, but are largely underreported to date. Over the past two decades, several sporadic human cases of CCHF occurred in countries such as Kenya [3], South Africa [9], Senegal [10,11], Mauritania [12], and Uganda [9,[13], [14], [15]]. Larger outbreaks were detected in Mauritania in 2003 (38 confirmed cases) and Sudan, with two successive epidemics in 2008 and 2009 [16,17].

Risk factors of CCHF infections included close contact with the fluid of infected animals and exposure to tick bites, which are particularly favored by low knowledge about the disease, and risky biosecurity practices in farms [7,18]. Most studies focus on the occupational angle of CCHF and other zoonotic diseases, targeting people at risk of the disease due to their profession [19,20]. In Burkina Faso, livestock farming and marketing are essential for the local economy and the daily income of rural populations [21]. Livestock practices are based on local breeds and produced mainly in extensive and semi-intensive systems, including mixed crop-livestock systems in agro-pastoral and pastoral systems with low biosecurity practices [22]. In these systems, livestock live in close proximity to their owners, hence exposing them to a high risk of being affected by a zoonotic disease. Therefore, the One Health approach that emphasizes the interconnectedness of human, animal, and environmental health is helpful for a better understanding of the dynamics of zoonotic diseases. The only and last confirmed human case in Burkina Faso was detected in 1984, [23] and despite the absence of recent cases of CCHF, the country is deemed a high-risk zone for the occurrence of the disease [9,24]. This recognition underscores the importance of proactively studying and understanding the factors influencing the potential emergence and spread of CCHF [1,25]. Among those factors, the knowledge, attitudes, and practices in rural communities that keep livestock toward zoonotic diseases, including CCHF, are not well explored. Our study aims to broadly assess the knowledge, attitudes, and practices of mixed crop-livestock farmers toward CCHF and zoonotic diseases. The results of this study will inform frameworks and tools to control CCHF and other emerging zoonotic diseases.

**Fig. 1:**
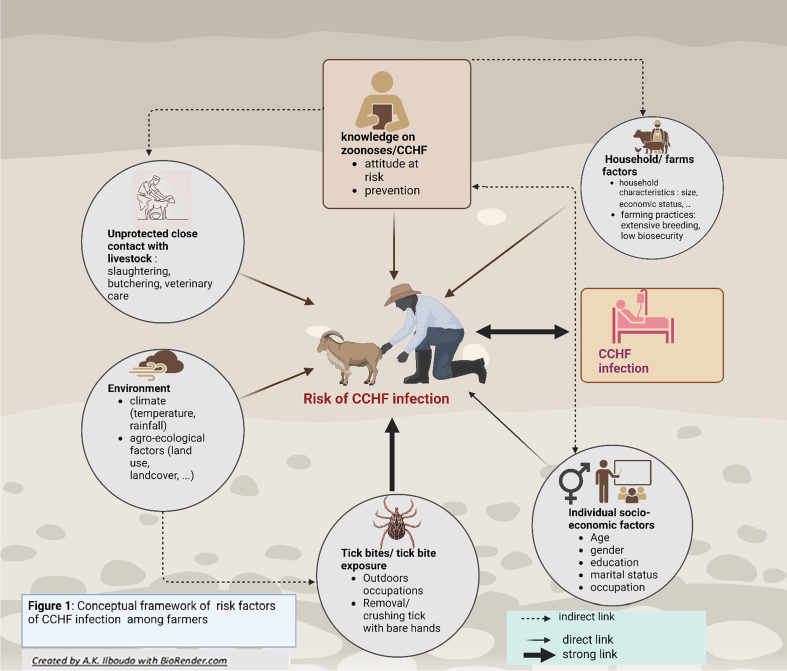
Conceptual framework of risk factors of CCHF infection among farmers.

***Conceptual framework of factors influencing knowledge, attitudes, and practices toward zoonoses and risks of CCHF in mixed crop-livestock farming*** (Fig. 1).

Practices in mixed crop-livestock farming can increase the risk of zoonotic diseases. Research shows that human behavior and socio-demographic factors, especially occupations like farming, slaughtering, hunting, and shepherding, which involve close contact with animals and their bodily fluids, contribute to the spread of diseases like CCHF. A meta-analysis from Nasirian *et al.*, [18] and other reviews [26,27] have demonstrated such interconnectedness. Additionally, exposure to tick bites in the context of external activities within a high-risk environment is a determinant of exposure, well described in many studies and reviews [9,28]. This environment, conducive to the presence and proliferation of ticks, is also a risk factor revealed in local and global studies [[29], [30], [31]]. Livestock plays a pivotal role in maintaining the virus as hosts for ticks; they develop a mild subclinical infection and can act as an amplifying host [32,33]. This same environment is contingent on individual factors such as age, gender, education, and household characteristics dependent on socio-economic status and contributing to the risk. All these factors directly or indirectly contribute to accessing knowledge that enables the adoption of livestock practices that are aligned to standard biosecurity guidelines [34,35]. A sound understanding of zoonoses, in general, is theoretically correlated with implementing preventive measures, such as wearing personal protective equipment during high-risk activities, safeguarding against zoonoses in general, and, to a lesser extent, CCHF [34,36,37].

## 2 Methods

### 2.1 Ethics statement

Before its implementation, the study was approved by the Burkina Faso National Committee of Health Research Review Board (**Ref: 2022–04-081**) and the Institutional Research Ethics Committee of the International Livestock Research Institute (**Ref: ILRI-IREC2022–15)**. Informed consent to participate in the study was obtained from all subjects and/or their parents or legal guardians. All methods were carried out following relevant guidelines and regulations following the Helsinki recommendations [38].

### 2.2 Description of the study sites

**Fig. 2:**
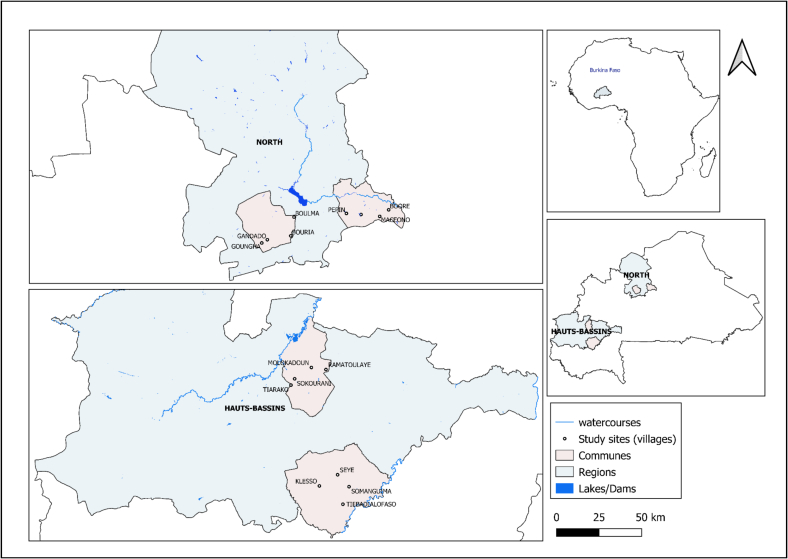
Situation map of the study sites.

The study was implemented in two regions (the “Hauts-Bassins” and the North regions) of Burkina Faso, a landlocked country in West Africa. It is a resource-limited country with a total population of more than 21 million (51.7 % female) (Fig. 2). Livestock production is one of the critical economic sectors of the country as it provides products and services to people, such as food and income [39,40].

The two regions were chosen for the following considerations: they share borders with Mali, where a CCHF outbreak occurred in 2020 in the region of Mopti [9], and these regions are the site of intensive cross-border transhumance of livestock [41].

The “Hauts-Bassins” region is a mixed farming-livestock system situated in the western part of the country in a South-Soudanian agro-climatic zone with an estimated human population of 2,238,375 in 2019, with 51.1 % females [42]. The North region is situated in the driest part of the country, with a semi-desert climate and phytogeographical zone. The total population of this region was 1,720,908 in 2019, with 52.2 % female [42]. Livestock production is predominantly extensive and semi-intensive [21]. From each of the two regions selected, two communes were purposefully chosen considering their accessibility and local farming practices. Four villages were then randomly selected from the list of villages in each commune considering the average population size for one village in the communes and the sample size targeted (sixteen villages in total).

### 2.3 Sample size estimation

The sample size was calculated using the standard formula for determining minimum number of subjects that are needed to estimate a popuation proportion (**Formula 1**). We assume that 50 % of farmers were at risk of CCHF considering the lack of specific similar recent reference studies in the region [43]. The formula used is:

(1) $$N=\frac{Z^{2}p\left(1-p\right)}{e^{2}}*D_{\mathrm{eff}}$$

N *is the sample size for a cross-sectional survey.*

*Z is 1.96 at a 95 % confidence level.*

*p is the estimated proportion of CCHF-positive individuals.*

*and e is the accuracy of the desired estimate. Its value is set at 10 %.*

**Table:** 

*Deff is the adjustment for design effect, it is fixed at 2.8* *The Deff is calculated using the formula:***Deff(DesignEffect) = 1** **+** **ICC(K-1)** *ICC = intra-cluster correlation fixed at 0.2, K = average cluster size fixed at 10*

This allows us to calculate a sample size of 283 persons for each of the two regions, i.e., **566 persons in total**, from the above formula when considering a proportion of potential loss of 5 %.

### 2.4 Study population and participant selection

The study focused on all individual males and females of six years old and above from rural settings who engaged in agro-pastoral activities. In each of the selected villages, a list of households was compiled through a preliminary census conducted by the study investigators. Eight to 11 households per village were randomly selected based on the size of the village.

To be selected, a household was expected to practice crop and livestock farming and possess a minimum of ten head of livestock (including large and small ruminants). Up to five consenting household members were interviewed individually.

### 2.5 Questionnaire design, validation, and data collection

Based on the available literature review, a questionnaire was developed to collect socio-demographic information from selected households and their members, along with specific questions related to agro-pastoral practices and the assessment of individual and collective risk regarding CCHF, as well as participants' knowledge of zoonoses (Supplementary Materials 2). The socio-demographic information and agro-pastoral activities of the households and their members primarily included details such as household size, the number of men, women, and children, cultivated land area, types and number of raised animals, household possessions (including livestock, cars, agricultural machinery, motorcycles). Additionally, individual data such as age, gender, and education level were collected. The questionnaire also incorporated specific items related to risky attitudes and practices regarding CCHF, as well as knowledge about zoonotic diseases, in line with the conceptual framework (Fig. 1). The questionnaire was tested during a pilot phase of the study on 5 % of the total sample size and then validated by the study team. The validation consisted of the identification of unclear questions for revision and adjustment after the pretest.

During the study, each selected participant who provided consent was interviewed face-to-face using a questionnaire administered through the Open Data Kit (ODK) [44] application on a mobile device. Prior to individual interviews, the head of the household was interviewed to collect information on household characteristics. For participants under the age of 18, socio-demographic data and information on attitudes and practices were collected from parents or legal guardians. The questionnaire related to knowledge of zoonoses was administered only to respondents aged 18 years and above. Each questionnaire was completed by a team comprising human, animal, and community health workers. This One Health approach was privileged to capture better the interactions between humans, animals, and the environment as outlined in the conceptual framework (Fig. 1).

### 2.6 Data quality control

To ensure uniformity, the data collection instrument was initially created in French, then translated into the most common local language spoken in the two regions (Dioula in the “Hauts-Bassins” and Mooré in the North), and subsequently translated back into French and English. A three-day training was provided to the data collectors and supervisors on the questionnaire and the data collection procedure. Additionally, the data collected were evaluated daily by supervisory personnel to ensure their completeness and accuracy.

### 2.7 Data management and analysis

#### 2.7.1 Variables definition

Our analysis focused on knowledge, attitudes, and practices related to zoonotic diseases in general, with a particular emphasis on CCHF.

- Two dependent variables were identified and estimated using the questionnaire variables:
✓ the attitudes and practices at risk score for CCHF,
✓ the knowledge score toward zoonoses.

The risky attitudes and practices for CCHF and the participant knowledge toward zoonoses were evaluated using the evaluation scales described in the Supplementary Material 1 **(**Table 1, Table 2, Table 3**)**.

✓ **Attitudes and practices at risk for CCHF**

The assessment of attitudes and practices at risk of exposure to CCHF was conducted by developing an evaluation scale. The conceptual framework (Fig. 1) was used to construct a scale based on a compilation of attitudes and practices recognized as risk factors for disease exposure derived from a literature review (Supplementary material **:** Table 1). A combination of binary answers questions (yes = 1 and no = 0) and a 3 to 5-point Likert scale was used in a series of 15 questions. The total score was calculated by summing the raw scores for the 15 questions ranging from 4 to 26, with a higher score overall indicating more risky attitudes and practices for CCHF (Supplementary material 1**:** Table 3).

✓ **The knowledge score toward zoonoses**

**Table 1:** Study population and household characteristics.

	*Household characteristics (N* *=* *717)*		
Variables	“Hauts-Bassins” region n (%)	Northern region n (%)	Total N (%)
**Age**			
<15 years	65(19.1)	81(21)	146(20.4)
15–34 years	129(37.8)	143(38)	272(37.9)
35 years and more	147(43.1)	152(40.4)	299(41.7)
**Gender**			
Male	205(60.1)	271(72)	476(66.4)
Female	136(39.9)	105(27.9)	241(33.6)
**Education**			
None	225(66)	256(68.1)	481(67.1)
Primary school	86(25.2)	89(23.7)	175(24.4)
secondary and university education	30(8.8)	31(8.2)	61(8.5)
**Marital status**			
Married monogamous	84(24.6)	93(24.7)	177(24.7)
Married polygamous	145(42.5)	117(31.1)	262(36.5)
Single/divorced/widowed	112(32.8)	166(44.1)	278(38.8)
**Communes**			
K. Vigue	166 (48.7)	–	166(23.2)
Satiri	175 (51.3)	–	175(24.4)
Yako	–	197(52.4)	197(27.5)
Tema-Bokin	–	179(47.6)	179(25)
**Household size**			
2–15 persons	141(41.3)	52(13.8)	193(26.9)
16–35 persons	173(50.7)	196 (52.1)	369(51.5)
36 persons and more	27 (7.9)	128 (34.0)	155(21.6)
**Number of men in the household**			
1–5 men	192 (56.3)	142 (37.8)	334 (46.6)
6 men and more	149 (43.7)	234 (62.2)	383 (53.4)
**Number of women in the household**			
1–5 women	205 (60.1)	112 (29.8)	317 (44.2)
6 women and more	136 (39.9)	264 (70.2)	400 (55.8)
**Number of children in the household**			
1–9 children	226(66.3)	112 (29.8)	338 (47.1)
10 children and more	115 (33.7)	264 (70.2)	379 (52.9)
**Farming surface**			
1–4 ha	101 (29.6)	163(43.4)	264 (36.8)
5 ha and more	240 (70.4)	213 (56.6)	453 (63.2)
**Household own the farmland**			
Yes	301 (88.3)	361 (96)	662(92.3)
No	40 (11.7)	15 (4)	55 (7.7)
**Household own the grazing areas**			
Yes	119(34.9)	356(94.7)	13 (14.1)
No	222(65.1)	356(94.7)	578(80.6)
**Minimum distance of livestock grazing area**			
< 1 km	97(28.4)	89(23.7)	186(25.9)
1–5 km	186(54.5)	279(74.2)	465(64.8)
6–10 km	27(7.92)	5(1.3)	32(4.5)
>10 km	31(9.1)	3(0.8)	34(4.7)
**Household have access to water**			
Yes	341(100)	346(92)	687(95.8)
No	0(0)	30(8)	30(4.2)
**Main source of water**			
Running water	0(0)	21(5.6)	21(2.9)
Common borehole	252(73.9)	278(73.9)	530(73.9)
Individual borehole	9(2.6)	10(2.7)	19(2.6)
well	80(23.5)	67(17.8)	147(20.5)
**Main source of power**			
National grid	7(2.3)	15(4.8)	22(3.5)
Own sources	304(97.7)	297(95.2)	601(96.5)
**Television in the household**			
Yes	176(51.6)	71(18.9)	247(34.4)
No	165(48.4)	305(81.1)	470(65.6)
**Radio in the household**			
Yes	261(7605)	323(85.9)	584(81.5)
No	80(23.5)	53(14.1)	133(18.5)
**Toilets**			
Yes	295(86.5)	316(84)	611(85.2)
No	46(13.5)	60(16.0)	106(14.8)
**Agricultural machine/ car in the household**			
Yes	75(22)	75(19.9)	150(20.9)
No	266(78)	301(80.1)	567(79.1)
**Motorcycle in the household**			
Yes	303(88.9)	316(84)	619(86.3)
No	38(11.1)	60(16)	98(13.7)
**Main fuel for used cooking**			
wood	270(76.2)	329(87.5)	599(83.5)
charcoal	71(20.8)	47(12.5)	118(18.5)
**Type of breeding**			
Extensive	330(96.8)	376(100)	706(98.5)
Semi-extensive	11(3.2)	0(0)	11(1.5)
**Main purpose of breeding**			
fattening (selling)	336(98.5)	376(100)	712(99.3)
dairy production	5(1.5)	0.0(0.0)	5(0.7)

**Table 2:** Univariable analysis of factors associated with CCHF attitudes and practices risk score and zoonoses knowledge score among farmers.

		Attitudes and practices at risk of CCHF N = 717		Zoonoses knowledge score N = 508	
Variables		Coefficient (95 % Confident interval)	p-value	Coefficient (95 % Confident interval)	p-value
**Region**					
Hauts-Bassins		1.41(0.82; 0.99)	<0.001***	0(base)	–
North		0(base)	–	0.76 (0.19; 1.34)	0.009**
**Gender**					
Male		2.31(1.7; 2.91)	<0.001***	1.59(1.01; 2.18)	<0.001***
Female		0(base)	–	0(base)	–
**Education**					
No education		0(base)	–	0(base)	–
Educated		−0.81(−1.43; −0.18)	0.012**	0.47(−0.18; 1.13)	0.161
**Marital status**					
Married monogamous		0(base)	–	0(base)	–
Married polygamous		0.1(−0.67;-0.87)	0.796	−0.04(−0.68;0.6)	0.904
Single/divorced/widowed		−0.83(−1.59; −0.07)	0.033	0.33(−0.56;1.23)	0.469
**Communes**					
K. Vigue		0(base)	–	0(base)	–
Satiri		1.68(0.84;2.52)	<0.001***	−0.72(−1.53;0.1)	0.084
Tema-Bokin		−0.21(−1.02;0.61)	0.617	0.24(−0.55;1.02)	0.556
Yako		−0.93(−1.76; −0.09)	0.029*	0.65(−0.2,1.5)	0.132
**Household own the farmland**					
Yes		−1.63(−2.74; −0.52)	0.004**	0.28(−0.81; 1.37)	0.615
No		0(base)	–	0(base)	–
**Household own the grazing areas**					
Yes		2.10 (1.37;2.84)	<0.001***	−1.29(−2.01; −0.57)	<0.001***
No		0(base)	–	0(base)	–
**Household have access to water source**					
Yes		2.19(0.72;3.67)	0.004**	−3.08(−4.37;-1.79)	<0.001***
No		0(base)	–	0(base)	–
**Main source of water**					
Running water		0(base)	–	0(base)	–
Common borehole		1.3(−0.47;3.06)	0.149	−2.68(−4.3;-0.96)	0.002**
Individual borehole		0.15(−2.36;2.67)	0.903	−3.3(−5.6;-0.97)	0.006**
well		0.79(−1.06;2.64)	0.403	−2.41(−4.2;-0.64)	0.008**
**Main source of power**					
National grid		0(base)	–	0(base)	–
Own sources (solar, …)		−1.93(−3.68;-1.18)	0.030**	0.79(−0.79;2.38)	0.325
**Television in the household**					
Yes		0.89(0.27;1.51)	0.005**	−0.37(−0.98;0.23)	0.225
No		0(base)	–	0(base)	–
**Radio in the household**					
Yes		−0.09(−0.85;0.67)	0.815	−0.5(−1.25;0.24)	0.182
No		0(base)	–	0(base)	–
**Toilets**					
Yes		0.79(−0.04;1.63)	0.062	0.55(−0.27;1.37)	0.187
No		0(base)	–	0(base)	–
**Agricultural machine/ car in the household**					
Yes		−0.08(−0.81;0.65)	0.832	−0.66(−1.38;0.05)	0.069
No		0(base)	–	0(base)	–
**Motorcycle in the household**					
Yes		1.12(0.26;1.98)	0.011*	−1.26(−2.12;0.41)	0.004**
No		0(base)	–	0(base)	–
**Main fuel for used cooking**					
wood		0(base)	–	0(base)	–
charcoal		−0.48(−1.28;0.32)	0.241	1.25(0.48;2.02)	0.001**
**Type of breeding**					
Extensive		0(base)	–	0(base)	–
Semi-extensive		3.73(1.33;6.13)	0.002**	−0.76(−2.97;1.43)	0.494
**Main purpose of breeding**					
fattening (selling)		0(base)	–	0(base)	–
dairy production		3.82(0.27;7.38)	0.035*	1.91(−1.02;4.84)	0.202
**Minimum distance of livestock grazing area**					
< 1 km		0(base)	–	–	–
1–5 km		1.19(0.5; 1.84)	<0.001***	0.21(−0.44;0.86)	0.534
6–10 km		3.54(3.11;5.98)	<0.001***	−1.76(−3.14;-0.37)	0.013*
>10 km		5.63(4.23;7.04)	<0.001***	−0.64(−2.11;0.81)	0.382
**Variables**		**Coefficiant (95 % Confident interval)**	**p-value**	**Coefficiant**	**95 % Confident interval**
**Age**		0.03(0.01;0.04)	0.001**	0.02(0.002;0.04)	0.024**
**Household size**		−0.005(−0.02;0.01)	0.426	−0.004(−0.002;0.01)	0.602
**Number of men in the household**		−0.06(−0.11;-0.01)	0.021*	0.078(−0.024;-0.13)	0.005**
**Number of women in the household**		−0.07(−0.12;-0.02)	0.003**	0.05(−0.007; 0.1)	0.022**
**Number of children in the household**		0.006(−0.02;0.03)	0.557	−0.04(−0.07;-0.02)	0.001**
**Farming surface (hectare)**		0.04(0.01;0.07)	0.011*	0.02(−0.01;0.05)	0.200
**Number of cattle raised in the household**		−0.004(−0.007;-0.000)	0.026*	−0.005(−0.008;-0.001)	0.004**
**Number of small ruminants (sheep and goats) raised in the household**		−0.004(−0.01;0.00)	0.083	0.05(−0.0006;0.1)	0.045*
**Number of asins in the household**		−0.002(−0.06;0.06)	0.927	−0.06(−0.01;0.1)	0.011*
**Number of poultry raised in the household**		−0.0004(−0.002;0.001)	0.618	−0.001(−0.002;0.0002)	0.098
**CCHF risk attitudes and practices score**		–	–	0.01(−0.05; 0.08)	0.694
**Knowledge of zoonoses score**		0.14(0.04;0.24)	0.007**	–	–

*P < 0.05; **P < 0.01; ***P < 0.001.

**Table 3:** Multivariable seemingly unrelated regression analysis of factors associated with attitudes and practices at risk of CCHF and knowledge toward zoonoses.

	Attitudes and practices at risk of CCHF N = 508		Zoonoses knowledge score N = 508	
Variables	Coefficient (95 % Confident interval)	p-value	Coefficient (95 % Confident interval)	p-value
**Intercept**	11.04(9.8; 12.25)	<0.001 ***	12.1(10.99; 13.18)	<0.001***
**Region**				
Hauts-Bassins	0(base)	–	0(base)	–
North	−1.1(−1.86;-0.28)	<0.008**	0.44(−0.22; 1.11)	0.192
**Gender**				
Male	2.85(2.14;3.56)	<0.001***	1.5(0.94;2.14)	<0.001***
Female	0(base)	–	0(base)	–
**Education**				
None	0(base)	–	0(base)	–
Formal education	−0.41(−1.19;-0.37)	0.309	0.32(−0.33;0.98)	0.336
**Household own the grazing areas**				
Yes	1.57(0.47;2.66)	0.005**	−0.57(−1.49;0.35)	0.229
No	0(base)	–	–	–
**Agricultural machine/vehicle in the household**				
Yes	−0.67(−1.59;0.23)	0.147	−0.6(−1.37;0.17)	0.127
No	0(base)	–	0(base)	–
**Minimum distance of livestock grazing area**				
< 1 km	0(base)	–	–	–
1–5 km	1.44(0.7; 2.2)	<0.001***	−0.21(−0.84;0.41)	0.510
6–10 km	3.06(1.46; 4.6)	<0.001***	−1.99(−3.34;-0.64)	0.004**
>10 km	4.45(2.66;6.24)	<0.001***	−0.43(−1.93;1.07)	0.571
**Age**	0.01(−0.01;0.03)	0.301	0.02(0.002;0.04)	0.028**
**Farming surface (hectare)**	0.02(−0.01;0.0362)	0.175	0.03(0.002;0.06)	0.032**
**Number of cattle raised in the household**	−0.003(−0.006;0.00)	0.036*	−0.003(−0.006;-0.000)	0.038*
**Household size**	0.003(−0.017;0.023)	0.766	−0.02(−0.04;-0.005)	0.009**

*P < 0.05; **P < 0.01; ***P < 0.001.

To assess knowledge of CCHF, we assumed at the outset of the study that farmers' knowledge of CCHF would be limited, given that the country had never officially recorded any active cases of the disease. Therefore, we developed a scale to evaluate knowledge of zoonoses in general, emphasizing hemorrhagic fevers and vector-borne zoonoses as a proxy of CCHF knowledge (Supplementary material 1**:** Table 2). The same combination of binary answers questions (yes = 1 and no = 0) and Likert scale questions was used in a series of 11 questions as the one form risk evaluation. A total knowledge score was calculated by adding up the raw scores for the 11 questions, ranging from 5 to 22, with higher scores and a higher overall score indicating more accurate knowledge (Supplementary material 1**:** Table 3).

### 2.8 Data management and analysis

The data entered on the phone were synchronized, extracted from the digital platform, and anonymized. Microsoft Excel® was used for tabulation and recording data, and STATA® 17 (StataCorp, College Station, TX, USA) was used for data cleaning, processing, and analysis.

#### 2.8.1 Descriptive and univariable analysis

A descriptive analysis of households, participants' characteristics, knowledge, attitudes, and practices was carried out. A bivariable analysis was conducted to find an association between the dependent variable (the attitudes and practices at risk for CCHF; the knowledge level of participants toward other zoonoses) and individual socio-economic and household characteristics variables. The Chi-square test assessed associations between two categorical variables, and the Pearson or Spearman correlation tests were used to measure the association between two quantitative variables. Ordinary Least squares (OLS) linear univariable regression was used to estimate the association between each outcome and the independent variables.

#### 2.8.2 Seemingly unrelated regression (SUR) model building

A Seemingly unrelated regression (SUR) [45] multivariable modeling was used in our study to account for the assumed correlation between the two independent variables: attitudes and practices toward CCHF and knowledge of other zoonoses. The interconnectedness between knowledge and behaviors, especially in livestock farmers, is well-documented in the literature [46,47]. Instead of considering knowledge, attitudes, and practices as separate entities, SUR integrates these components into the analysis, allowing a holistic comprehension of how knowledge can influence attitudes and practices and vice-versa. SUR is known to provide more efficient parameter estimates when the error terms of the equations are correlated. Since there is a high probability that the knowledge, attitudes, and practice components are correlated or influence each other, using SUR could produce more accurate estimates than estimating separate equations for each component. This method can also provide more accurate and robust estimates accounting for the shared variability or common factors influencing the outcomes [48,49].

By using the SUR approach, the parameters of all the equations are jointly estimated, allowing each equation, which may contain a set of independent variables distinct from the others, to consider the data provided by the other equations. The equations of the SUR are as follows [50]:

(2) $$S_{\mathrm{ij}}=f\left(X_{i}\right)$$

j stands for the knowledge score, attitudes, and practices score (j = 1, 2, and 3). Sij is the farmer's KAP score; Xi stands for the socio-economic and demographic variables related to farmers, i = 1,2, …n.

Eqn 2 can be converted into the following Eqs. [50]:

(3) $$\left\{\begin{array}{c} S_{1i}=\alpha_{1}+\sum \beta_{1n}+X_{\mathrm{ni}}+\mu_{1i}\\ S_{2i}=\alpha_{2}+\sum \beta_{2n}+X_{\mathrm{ni}}+\mu_{2i} \end{array}\right)$$

S1i is the equation, α stands for the intercept, β for the coefficients, and μ for the residuals, Xi stands for the covariates related to farmers and household characteristics, i = 1,2,3…n.

To build the SUR model, variables with a *p*-value ≤0.2 in the univariable OLS regression were entered into the seemingly unrelated regression model after checking and correcting multicollinearity using variance inflation factors calculation (VIF). A forward stepwise elimination method was used to obtain the final model choosing the model with the lowest calculated Akaike's information criterion (AIC). Coefficients with a 95 % confidence interval were calculated to measure the strength of the association between the outcomes and independent variables. The level of significance was set at *p* < 0.05. The final model obtained was validated using the different validation tests available for SUR models in STATA® version 17: Breusch-pagan test of independence, marginal means, and the Wald tests of composite linear hypotheses.

## 3 Results

### 3.1 Socio-demographic characteristics of the respondents

Overall, 717 farmers from four communes were included in our study. Notably, 20.4 % (146/717) of participants were under 15 years old, while 41.7 % (299/717) were 35 years or older. The majority were male (66.4 %, 476/717), and a substantial proportion (67.1 %, 481/717) had no formal education. Household characteristics indicated that 51.5 % (369/717) lived in households with 16 to 35 people, and more than half of these households (55.8 %) had over six women. Regarding livestock rearing practices, 98.5 % (706/717) practiced extensive rearing, and 99.3 %(712/717) stated that fattening was the main objective of rearing (Table 1).

### 3.2 Knowledge attitudes and practices regarding CCHF

Concerning attitudes and practices at risk of CCHF infection, our analysis revealed that farmers were in regular contact with their animals, with 45.3 % (325/717) having contact time estimated at 6 to 12 h daily. The primary animals of contact were cattle (79.2 %) and small ruminants (68.1 %). Notably, only 1.7 % (12/717) of farmers regularly used personal protective equipment (PPE) during high-risk contacts. Contacts with animal body fluids mainly occurred through slaughtering with bare hands (17.3 %), butchering (14.4 %), and assisting with calving (14.4 %). Among those with a history of tick bites, 75.5 % (313/409) practiced manual removal of ticks, while 28.1 % practiced ticks manual crushing (**Supplementary material:** Table 1).

The knowledge score was evaluated among adult respondents (age ≥ 18 years: *n* = 508). In terms of respondents' knowledge toward other zoonoses, 58.7 % (298/508) of farmers have ever heard of zoonotic diseases in their language, while 28.4 % (144/508) could correctly name at least three different zoonotic diseases in their language. A total of 40.9 % (208/508) of the respondents were able to name at least three different ways of zoonotic disease transmission, and 53.5 % (272/508) of them could name three different methods of prevention (**Supplementary material:** Table 2).

### 3.3 Factors associated with knowledge toward zoonoses and attitudes and practices at risk for CCHF

The mean+/− SD scores of respondents for their attitudes and practices at risk for CCHF and knowledge of zoonotic diseases were 12.60±4.04 (smallest =5; largest = 26) and 13.3±3.33 (smallest = 5, largest = 22) respectively (**Supplementary material:** Table 3).

#### 3.3.1 Univariable analysis using OLS linear regression

Univariable analysis conducted using OLS linear univariate regression showed a gender-oriented association of both outcomes (knowledge on zoonoses, and attitudes and practices at risk of CCHF). Males showed a higher knowledge score (Coef [95 %CI] = 0.76[0.19;1.34]; *p*-value<0.001) but also had a higher risk score of CCHF (Coef [95 %CI] = 2.31[1.7; 2.91]; *p*-value<0.001). Additionally, as age increases, both risky attitudes and practices for CCHF (Coef [95 %CI] = 0.03[0.01;0.04]; p-value<0.001) and knowledge of zoonoses (Coef [95 %CI] = 0.02[0.002;0.04]; p-value = 0.024) increased.

Regional disparities were also observed: respondents from the “Hauts-Bassins” region exhibited higher risky attitudes and practices toward CCHF (Coef [95 %CI] = 1.41[0.82;0.99]; p-value<0.001), while those from the North region had better knowledge scores (Coef [95 %CI] = 0.76[0.19;1.34]; *p*-value = 0.009). Several household socio-economic factors were associated with CCHF risk. Owning a farm or grazing areas was associated with increased as was practicing semi-intensive livestock rearing (vs extensive) (Coef [95 %CI] = 3.73[1.33;6.13]; *p*-value = 0.002) and milk production (vs livestock fattening) (Coef [95 %CI] = 3.82[0.27;6.13]; *p*-value = 0.035). Conversely, the number of women and adult men in the household was negatively associated with CCHF risk. Respondent age (Coef [95 %CI] = 0.03[0.01;0.04]; p-value<0.001), ownership of grazing areas (Coef [95 %CI] = 2.1[1.37;2.84]; p-value<0.001) and the number of children in the household were positively associated with attitudes at risk for CCHF (Table 2).

#### 3.3.2 Seemingly unrelated regression (SUR) modeling

Multivariate analysis using the SUR modeling confirmed that the knowledge, attitudes, and practices regarding other zoonoses and CCHF are gender-oriented. Men have higher odds of attitudes and practices at risk of CCHF (Coef [95 %CI] = 2.85[2.14;3.56]; *p*-value<0.001) but paradoxically had a better score in terms of knowledge about zoonoses (Coef [95 %CI] = 1.5[0.94;2.14]; p-value<0.001). Furthermore, our model revealed a statistically significant association between age and knowledge scores, indicating that as respondents' age increased, their understanding of zoonoses also increased (Coefficient [95 % Confidence Interval] = 0.02 [0.032; 0.055]; p-value = 0.028).

The distance from the household to the livestock grazing area was associated with the risk of CCHF for the farmers. The risk of CCHF grows with the distance. For instance, compared to households with a grazing distance of less than one (01) km, households with grazing areas more than 10 km away have a 4.45 point higher CCHF risk score ([95 %CI] = [2.66;6.24]; p-value <0.001). Indicators of the household's socio-economic status, such as the cultivated area, were associated with better zoonoses knowledge scores (Coef[95 %CI] = 0.03[0.002;0.06]), whereas owning the livestock grazing area increased the CCHF risk score (Coef [95 %CI] = 1.57[0.47;2.66]; p-value = 0.005) (Table 3).

## 4 Discussion

Our study aimed to analyze knowledge, attitudes, and practices regarding zoonoses, among rural mixed crop-livestock farmers with a focus on CCHF, and to identify their key drivers. The findings provide insights into the risk factors associated with CCHF and underscore the importance of understanding the complex relationship between human behaviors, livestock-rearing practices, and zoonotic disease risk. The study also revealed the high level of attitudes and practices associated with CCHF risk while knowledge about other zoonoses was limited. We used the Seemingly Unrelated Regression (SUR) approach in our analyses, allowing us to refine our predictive models capable of incorporating interactions between the two outcomes. This allowed us to identify socio-demographic factors such as age, gender, and residence as determinants of farmers' behavior at risk of CCHF and their knowledge of zoonoses. Furthermore, household socio-economic conditions and certain agro-pastoral practices and activities were found to be predictors of both outcomes.

Our study highlights a low level of biosecurity applied among mixed crop-livestock farmers. Critical measures, such as the use of personal protective equipment (PPE) during high-risk activities, were almost non-existent (1.6 %), even though these practices were common. The overwhelming majority (98.5 %) were practicing extensive farming, with fattening considered to be the main objective for 99.3 % of livestock rearing. These practices, deeply anchored in the local agricultural context, contribute to the complex network of interactions between humans and animals, laying the foundations for the transmission of zoonotic diseases. The low implementation of biosecurity measures on smallholder farms has also been described in studies carried out in Tanzania [36], South Africa [51], and Ghana [52]. The poor accessibility of PPE for farmers combined with insufficient exposure to awareness messages may have contributed to the poor application of zoonoses prevention measures.

The study also shows a high proportion of farmers with high-risk contact with livestock body fluids. Practices such as slaughtering and butchering with bare hands, as well as high-risk behaviors during tick bites, such as manual removal and/or crushing, were common. These behaviors are widely recognized in the literature as key factors for exposure to CCHF, especially in at-risk professionals. In a meta-analysis, Nasirian *et al.* [18] identified proximity to and contact with the fluids of at-risk animals and exposure to tick bites as risk factors for CCHF infection. Amateur slaughter and butchering of animals are also common among non-professionals in communities during traditional and religious rites, further increasing the risk of CCHF infection. This underscores the need for targeted interventions including regulation of these amateur slaughter practices and dissemination of awareness messages about zoonosis prevention and best animal husbandry practices to minimize the occurrence of CCHF and other zoonoses [53,54]. Notably, these messages and prevention measures will be beneficial not only for CCHF but also for other zoonotic diseases with similar transmission modes, as they may help reduce biosafety-risk practices in general.

The overall knowledge of farmers about zoonoses was limited. Paradoxically, while barely a third of respondents had ever heard of zoonoses, more than half of them could name three routes of transmission of animal diseases and prevention measures. These findings are consistent with those reported in South Africa [51], Uganda [36] and Turkey [55]. The results highlight the importance of improving health awareness programs tailored to the local context, as described by Mangesho *et al.* in a qualitative study in Tanzania [56].

In our study, the Seemingly Unrelated Regression (SUR) modeling [45] was preferred over standard OLS regression for the multivariable analysis of factors associated with knowledge of zoonoses and attitudes and practices toward zoonoses and CCHF. OLS regression is commonly used for model prediction in medical studies when dealing with several related outcome variables [49]. However, it estimates several equations separately without considering the potential correlation between the models [50]. SUR methods provide a more nuanced understanding of the complex relationships within the study population and are frequently used in economics studies [57]. The primary motivations for using SUR in predicting attitudes and practices are its ability of estimating the knowledge model and the attitudes and practices models simultaneously while accounting for correlations between these dimensions and its capacity to provide efficient estimates of coefficients and standard errors by accounting for such correlations [49,58,59]. This technique could benefit from more extensive application in health and social sciences, notably in studies exploring complex interactions, such as those examining patient behaviors and knowledge, treatment adherence, and socio-demographic determinants [50].

The results of the multivariable SUR analysis confirmed the gender-based orientation of the farmers' behaviors and knowledge of zoonoses already shown by the OLS linear univariable regression. Men were found to be more exposed to the risk of CCHF while paradoxically demonstrating better knowledge scores about zoonoses. These results indicate the solid socio-cultural constraints in the communities studied, where women are confined to domestic and agricultural activities and are less involved in large ruminant breeding, mainly the preserve of men [60,61]. Women's livestock activities are more oriented to small ruminants and poultry. The poor education of women, their weak presence in forums for discussion and awareness-raising, and their low access to resources could explain their limited knowledge of zoonotic diseases and prevention measures [62,63].

In addition, our analysis revealed that greater distances traveled by livestock for grazing were associated with higher risk scores for CCHF. This result emphasizes the risk associated with livestock movement for zoonoses. Shepherds practicing transhumance can be exposed to various risks of zoonotic diseases due to poor hygienic conditions during migration, such as tick bites and inadequate feeding [64].

Farmer age was positively associated with knowledge scores, and attitudes and practices at risk increased with age. This suggests that high-risk practices and activities, such as slaughtering and butchering animals, may be socially reserved for adults or older individuals which could explain the rise in risk with age. The negative association between the household size and the knowledge score in our study reinforced this interpretation. Singh *et al.* reported a negative association between knowledge of zoonoses and age in India [66], reinforcing the idea of the influence of the local context on these covariates.

Socio-economic indicators, such as the size of the cultivated area and the number of cattle in the household, were associated with lower risk and/or better knowledge scores in the SUR multivariable model. The economic status remains an essential determinant of exposure to and risk of zoonoses, including CCHF. This was difficult to perceive and even nuanced with some indicators in our study, given the relative homogeneity of our study population and the regional disparities observed. However, the close relationship between household economic status, regional inequality, and the risk of exposure to CCHF and zoonoses is also described in studies in Tanzania [53], Pakistan [37], and Kenya [65]. This confirms the need for policymakers to consider socio-economic and local contexts when designing interventions.

Our study has certain limitations. Since it was conducted in communities where CCHF is not well-known, which may have affected the accuracy of the information collected and the expected parameter estimates of the risk factors being studied could be biased to the null. The absence of qualitative data limits our ability to capture the deeper social, cultural, and economic factors that may influence knowledge, attitudes, and practices. A qualitative approach would provide a more nuanced understanding of the underlying factors influencing knowledge attitudes and practices, considering intra-group specificities. Incorporating qualitative methods, such as interviews or focus group discussions, in future research could provide a more comprehensive understanding of these underlying factors.

## 5 Conclusion

In conclusion, our study offers valuable insights into the KAP of farmers regarding CCHF and other zoonoses in Burkina Faso. Applying SUR techniques enhances our analysis's precision, uncovering nuanced associations between socio-demographic factors and disease-related behaviors and knowledge, which should be promoted in similar studies. Our findings underscore the importance of context-specific interventions to mitigate the risk of zoonotic diseases and strengthen community resilience in the face of emerging health threats. Future research should further explore the dynamics of zoonotic diseases, incorporating longitudinal and qualitative studies and community-based interventions to inform sustainable strategies for disease prevention and control in the region.

## CRediT authorship contribution statement

**Abdoul Kader Ilboudo:** Writing – review & editing, Writing – original draft, Visualization, Software, Methodology, Investigation, Formal analysis, Data curation, Conceptualization. **Michel Dione:** Writing – review & editing, Writing – original draft, Project administration, Methodology, Funding acquisition, Conceptualization. **Ard M. Nijhof:** Writing – review & editing, Validation, Supervision, Conceptualization. **Martin H. Groschup:** Writing – review & editing, Validation, Supervision. **Ousmane Traoré:** Software, Formal analysis. **Guy S. Ilboudo:** Writing – review & editing, Writing – original draft, Investigation. **Zekiba Tarnagda:** Writing – review & editing, Conceptualization. **Madi Savadogo:** Writing – review & editing, Conceptualization. **Bernard Bett:** Writing – review & editing, Writing – original draft, Validation, Supervision, Project administration, Methodology, Funding acquisition, Conceptualization.

## Ethical approval

The ethics statement is detailed in the Methods section (2.1) of the text.

## Funding

The authors wish to thank the Federal Ministry for Economic Cooperation (BMZ), Germany, which funded this study as part of the applied research activities that were being implemented under the One Health Research Education and Outreach Centre (OHRECA). Additional funding was obtained from the CGIAR One Health Initiative, “Protecting human health through a One Health approach” that is supported by contributors to the CGIAR Trust Fund (https://www.cgiar.org/funders/)”.

## Declaration of competing interest

The authors declare that they have no known competing financial interests or personal relationships that could have appeared to influence the work reported in this paper.

## Data Availability

The datasets generated and/or analyzed during the current study are available from the corresponding author on reasonable request.

## Appendix A Supplementary data

Supplementary material 1 Image 1

Supplementary material 2 Image 2
