# Supplementary material for: Factors associated with knowledge, attitudes, and practices of mixed crop-livestock farmers on Crimean-Congo hemorrhagic fever (CCHF) and other zoonoses in Burkina Faso
Source: One Health. 2025 May 8;20:101066. doi: 10.1016/j.onehlt.2025.101066 (PMC12142541; doi:10.1016/j.onehlt.2025.101066)
Supplement: Supplementary file 1 — Supplementary material 1 [file mmc1.docx]

**Supplementary Materials 1**

**Table 1 :** Evaluation scale and classification of risky attitudes and practice toward zoonoses and CCHF

|  | **Variables** | **Answers and scores** | **Frequency n (%)**  **N=717** |
| --- | --- | --- | --- |
|  | **Duration of contact* with the animals** | <1h/day= 1 point | 268 (37.38) |
|  |  | 1-6h/day= 2  points | 44(6.14) |
|  |  | 6-12h/day =3 points | 325(45.33) |
|  |  | >12h/day=4 points | 80(11.16) |
|  | **Type of contact* with the animal** | Body care = 1 point | 341(47.6) |
|  |  | Veterinary care = 1 point | 84(11.7) |
|  |  | Cleaning of pens= 1 point | 435(60.67) |
|  |  | Milking = 1 point | 104(14.5) |
|  |  | Animal feeding= 1 point | 575(80.2) |
|  |  | Animal slaughtering = 1 point | 56(7.81) |
|  |  | Other circumstances = 1 point | 17(2.37) |
|  | **Type of contact* animal** | Cattle = 1 point | 568(79.22) |
|  |  | Goats/sheep=1 point | 488(68.06) |
|  |  | Asin= 1 point | 117(16.32) |
|  | **Usage of PPE during contact* at risk with animal** | Never = 4 points | 461(64.30) |
|  |  | Rarely=3 points | 235(32.78) |
|  |  | Often=2 points | 12(1.67) |
|  |  | Always = 1 point | 9(1.26) |
|  | **Transport livestock without PPE**** | Yes =1 point | 102(14.32) |
|  |  | No/Don't know=0 point | 615(85.77) |
|  | **Clean livestock pens** | Yes =1 point | 558(77.82) |
|  |  | No=0 point | 159(22.18) |
|  | **Give veterinarian care to animals** | Yes =1 point | 111(15.48) |
|  |  | No=0 point | 606(84.52) |
|  | **Milk cows/goats** | Yes =1 point | 119(16.60) |
|  |  | No=0 point | 598(83.40) |
|  | **Skin with bare hands** | Yes =1 point | 149(20.78) |
|  |  | No=0 point | 568(79.22) |
|  | **Helps to deliver animals with bare hands** | Yes =1 point | 103(14.37) |
|  |  | No=0 point | 614(85.63) |
|  | **Slaughter animals with bare hands** | Yes =1 point | 124(17.29) |
|  |  | No=0 point | 593(82.71) |
|  | **Touch dead animal carcasses with bare hands** | Yes =1 point | 160(22.32) |
|  |  | No=0 point | 557(77.68) |
|  | **Remove ticks with bare hands** | Yes =1 point | 344(47.98) |
|  |  | No/no tick bite history =0 point | 373(52.02) |
|  | **Crush tick with bare hands** | Yes =1 point | 163(22.73) |
|  |  | No=0 point | 554(77.27) |

* We define a contact as a direct interaction within a distance of 1 meter without wearing personal protective equipment

**PPE: personal protective equipment

**Table 2:** Evaluation and classification of participant knowledge toward zoonoses

|  | **variables** | **Answers and scores** | **Frequency n (%)**  **N=508** |
| --- | --- | --- | --- |
|  | Has ever heard of zoonoses | Yes =1 point | 298(58.7) |
|  |  | No=0 point | 210(41.3) |
|  | Think that animals can transmit diseases to human | Yes =1 point | 333(65.5) |
|  |  | No=0 point | 175(34.4) |
|  | Able to cite three different zoonoses | No=0 point | 364(71.6) |
|  |  | Yes = 1 point | 144(28.4) |
|  | Able to cite three ways of zoonoses transmission | No=0 point | 300(59.1) |
|  |  | Yes = 1 point | 208(40.9) |
|  | Able to cite three ways of preventing zoonoses | No=0 point | 236(46.5) |
|  |  | Yes = 1 point | 272(53.5) |
|  | All animal diseases can also affect humans | Strongly agree or Agree=0 point | 316(62.2) |
|  |  | Neither agree nor disagree= 1points | 132(26) |
|  |  | Disagree =2 points | 30(5.9) |
|  |  | Strongly disagree=3 points | 30(5.9) |
|  | I can be infected by animal diseases by cleaning their pens | Strongly agree =0 point | 12(2.3) |
|  |  | Agree=1point | 26(5.1) |
|  |  | Neither agree nor disagree= 2points | 143(28.2) |
|  |  | Disagree =3 points | 235(46.3) |
|  |  | Strongly disagree=4points | 92(18.1) |
|  | I can be infected by animal diseases during care delivery | Strongly agree =4 points | 98(19.3) |
|  |  | Agree = 3 points | 230(45.3) |
|  |  | Neither agree nor disagree= 2points | 154(30.3) |
|  |  | Disagree =1 points | 19(3.7) |
|  |  | Strongly disagree=0 points | 7(1.4) |
|  | I cannot be infected by manipulating the carcass of dead animals with bare hands. | Strongly agree or Agree =0 point | 234(46.1) |
|  |  | Neither agree nor disagree= 1points | 199(39.2) |
|  |  | Disagree =2 points | 53(10.4) |
|  |  | Strongly disagree=3 points | 22(4.3) |
|  | I can be infected by an animal disease even if I am not in contact with a sick animal | Strongly agree =0 point | 33(6.5) |
|  |  | Agree = 1points | 66(13) |
|  |  | Neither agree nor disagree= 2points | 193(38) |
|  |  | Disagree =3points | 144(28.3) |
|  |  | Strongly disagree= 4 points | 72(14.2) |
|  | An apparently healthy animal cannot transmit disease to humans | Strongly agree /Agree =0 point | 174(34.2) |
|  |  | Neither agree nor disagree= 1points | 222(43.7) |
|  |  | Disagree =2 points | 62 (12.2) |
|  |  | Strongly disagree=3 points | 50(9.8) |

**Table 3:** Summary of the outcomes variables attitudes and practices at risk of CCHF and knowledge toward other zoonoses

| **Variables** | **Characteristics** |
| --- | --- |
| **Attitudes and practices at risk of CCHF** | **N=717**  **Mean (+/-SD*) =** 12.6(+/- 4.04)  **Minimum score = 4**  **Maximum score = 26** |
|  | - **Intermediate and high attitudes and practices at risk (score≥12)=** 48.1%(345/717) - **Low attitudes and practices at risk (score <12)=** 51.9%(372/717) |
| **Knowledge toward zoonoses** | **N=508**  **Mean (+/-SD*) =** 13.3(+/- 3.3)  **Minimum score =** 5  **Maximum score =** 22 |
|  | - **Good knowledge toward zoonoses (score≥14)=** 52.2% (265/508) - **Limited knowledge toward zoonoses (score<14)=** 47.8% (243/508) |

***SD :** standard deviation
