# Supplementary material for: Factors associated with knowledge, attitudes, and practices of mixed crop-livestock farmers on Crimean-Congo hemorrhagic fever (CCHF) and other zoonoses in Burkina Faso
Source: One Health. 2025 May 8;20:101066. doi: 10.1016/j.onehlt.2025.101066 (PMC12142541; doi:10.1016/j.onehlt.2025.101066)
Supplement: Supplementary file 2 — Supplementary material 2 [file mmc2.docx]

**Supplementary Materials 2 : Questionnaires**

| ***Part* I: Household/Concession Questionnaire** | |
| --- | --- |
| Concession identification number | I__I__I__I - I__I__I__I |
| Household/concession characteristics | |
| Name of the village |  |
| Name of head of household |  |
| Head of household telephone | I__I__I_ I__I__I__I __I__I  I__I__I_ I__I__I__I __I__I |
| GPS coordinates of the concession | Latitude /________________ longitude______________________/ |
| Number of households in the concession | I__I__I__I |
| Number of individuals in the concession | I__I__I__I |
| Number of women | I__I__I__I |
| Number of adult males | I__I__I__I |
| Number of children in the concession/ household (<15 years) | I__I__I__I of which I__I__I girls and I__I__I boys |
| Number of houses in the concession | Of which I__I__I__I built with bricks and I__I__I__I built with earth |
| Practice agriculture | 🞏 Yes 🞏 No |
| The concession owns its own land (agriculture) | 🞏 Yes 🞏 No |
| The concession has its own grazing areas | 🞏 Yes 🞏 No |
| Water source of the concession | 🞏 Individual borehole 🞏 Common borehole 🞏 Pond/stream  🞏 Others................... |
| Power source | 🞏 Yes / national grid 🞏 Yes / own grid (e.g. solar, batteries) 🞏 No |
| Mobile phone in the household | 🞏 Yes .........(number) 🞏 No |
| Television | 🞏 Yes 🞏 No |
| Radio | 🞏 Yes 🞏 No |
| Toilets | 🞏 Yes 🞏 No |
| Agricultural machine or vehicle | 🞏 Yes 🞏 No |
| Motorcycle | 🞏 Yes 🞏 No |
| Fuel used for cooking | 🞏 Wood 🞏 Charcoal 🞏 Butane 🞏 Other.......................(specify) |
| Types and numbers of animals raised on the concession | 🞏 Cattle ........ 🞏 Goats............. 🞏 Sheep........ 🞏 Asins........ 🞏 Poultry (chicken, guinea fowl, duck, etc.) ........heads  🞏 other_______________________……..heads |
| Main objective of the breeding | 🞏 sale (meat) 🞏 sale (milk) 🞏 family 🞏 consumption (meat, milk) |
| Type of breeding | 🞏 mixed farming-livestock system 🞏 semi-intensive 🞏intensive |

| **Part II: Questionnaire for household participants** | | |
| --- | --- | --- |
| 1. Identification number of the herd(s) in contact with the participant (if sampled) | I__I__I__I - I__I__I__I  I__I__I__I - I__I__I__I  I__I__I__I - I__I__I__I | |
| 1. Identification number of the contact animal   (if only one animal identified) | I__I__I__I - I__I__I__I | |
| 1. Participant identification number | I__I__I__I - I__I__I__I - - I__I__I__I - I__I__I__I | |
| **SOCIO-DEMOGRAPHIC INFORMATION** | | |
| 1. Name |  | |
| 1. First name(s) |  | |
| 1. Sex | 🞏 Male 🞏 Female | |
| 1. Date of birth | I__I__I/ I__I__I/ I__I__I__I | |
| 1. Age (in years if more than 1 year in months if less than 1 year) | I__I__I__I years / I__I__I__I months | |
| 1. Telephone number of participant or parent/guardian if minor | /______________________________________/  ______________________________________/ | |
| 1. City/ village | /______________________________________/ | |
| 1. Area of residence | /______________________________________/ | |
| 1. Level of study | 🞏 never went to school 🞏 primary education 🞏 secondary school 🞏 6 high school | |
| 1. Marital status | 🞏1 = Married (monogamous) 🞏4 = Divorced/separated  🞏2 = Married (polygamous) 🞏3 = Widow/Widower  🞏5 = Single / unmarried 🞏6 = Other (specify)…….. | |
| 1. GPS coordinates (residence, sampling location) | Latitude /________________ longitude______________________/ | |
| 1. Recently moved | 🞏 Yes 🞏 No | |
| 1. If yes, place of origin and distance from current residence | /___________________________________/ distance __________/Km | |
| 1. Profession/ Occupation | /______________________________________/ | |
| 1. Duration in the profession | /_______/Years | |
| 1. Place of the practice of the occupation at risk | 🞏Farm Slaughterhouse 🞏livestock market 🞏 transhumance 🞏butchery  🞏 veterinary/medical staff 🞏 Others_____________________ | |
| 1. If farm or market, what type of animals are there? | 🞏Cattle 🞏Goats 🞏Sheep 🞏Donkey 🞏Poultry (chicken, duck,..)  🞏Others_______________________ | |
| **CLINICAL INFORMATION** | | |
| **Medical history** | | |
| 1. Obesity | 🞏 Yes 🞏 No | |
| 1. Cancer | 🞏 Yes 🞏 No | |
| 1. Diabetes | 🞏 Yes 🞏 No | |
| 1. HIV/other immune deficiency | 🞏 Yes 🞏 No | |
| 1. Heart disease | 🞏 Yes 🞏 No | |
| 1. HBP | 🞏 Yes 🞏 No | |
| 1. Sickle cell disease | 🞏 Yes 🞏 No | |
| 1. Pregnancy | 🞏 Yes 🞏 No | |
| 1. If yes, specify the quarter | 🞏 First trimester 🞏 Second trimester 🞏 Third trimester | |
| 1. History of febrile illness with bleeding | 🞏 Yes 🞏 No | |
| 1. Malnutrition | 🞏 Yes 🞏 No | |
| 1. Other pre-existing condition(s) | .....................................................................................................................(specify) | |
| **INFORMATION ON EXPOSURE FACTORS** | | |
| 1. How much time do you spend in contact (less than one meter or direct contact) with the animal per day? | 🞏Less than one hour 🞏1-6h 🞏6-12H 🞏>12h/day | |
| 1. Type/Circumstances of contact with animals | 🞏Body care 🞏Veterinary care 🞏Milking 🞏cleaning of pens  🞏Slaughtering/butchering 🞏Abattage/dépeçage 🞏embouche 🞏Autres_________________________(préciser) | |
| 1. What types of animals do you come into contact with the most? | 🞏cattle 🞏sheep 🞏goats 🞏others ________________________(specify) | |
| 1. Use of personal protective equipment during close contact with animal | 🞏never🞏 rarely 🞏often 🞏always | |
| 1. If yes, what PPE do you use? | 🞏Gloves 🞏Mask 🞏gown/working gear 🞏 Goggles 🞏 glasses  🞏 autres(préciser)___________________ | |
| 1. If transhumance or semi-extensive farming | Average distance travelled per day  🞏 less than 1 km 🞏1- 5 km 🞏6-10 km 🞏more than 10 km | |
| 1. Do you sleep with or near animals? | 🞏 Yes 🞏 No | |
| 1. Do you manually remove ticks from animals | 🞏 Yes 🞏 No | |
| 1. Contact with a sick person/animal in the last 30 days? | 🞏 Yes 🞏 No | |
| 1. If yes, please specify: | /__________________________________/ | |
| 1. Notion of tick bites | 🞏 Yes 🞏 No 🞏 Don't know | |
| **Signs & Symptoms (Currently and over the past 14 days)** | | |
| 1. Signs and symptoms of infection in the two weeks prior to the survey | **Clinical signs** |  |
|  | No | 🞏 Yes 🞏 No |
|  | Fever | 🞏 Yes 🞏 No |
|  | Curvatures | 🞏 Yes 🞏 No |
|  | Vomiting | 🞏 Yes 🞏 No |
|  | Diarrhea | 🞏 Yes 🞏 No |
|  | Cough | 🞏 Yes 🞏 No |
|  | Bleeding (non-traumatic) | 🞏 Yes 🞏 No |
|  | Dyspnea | 🞏 Yes 🞏 No |
| 1. Other (specify) |  | ____________________________  ____________________________  ___________________________ |
| 1. If signs, specify treatment(s) or management modalities |  | ___________________________ |
| **INFORMATION ABOUT THE SAMPLING** | | |
| 1. Sample taken | 🞏 Yes 🞏 No | |
| 1. Date of collection | I__ I __ I / I__I__I/ I__I__I__I__I | |
| 1. Conditions of preservation of the samples | 🞏Not adequate 🞏Adequate | |
| 1. Date sent to the laboratory | I__ I __ I / I__I__I/ I__I__I__I__I | |

| **Knowledge, Attitudes and Practices Questionnaire** | |
| --- | --- |
| **Knowledge** | |
| **zoonoses in general** | |
| 1. Are your animals affected by any diseases? | 🞏 Yes 🞏 No 🞏 Don't know |
| 1. Can you name three diseases that most often affect your animals? | a_________________________________________________🞏1 true 🞏2false  b_________________________________________________🞏1true🞏2false c__________________________________________________ 🞏1 true🞏 2false |
| 1. Have you ever heard of zoonoses (translate into local language) | 🞏 Yes 🞏 No |
| 1. Do you think that diseases from animals can be transmitted to humans? | 🞏 Yes 🞏 No 🞏 Don't know |
| 1. If YES, name three animal diseases that can be transmitted to humans | a_________________________________________________🞏1 true 🞏2false  b_________________________________________________ 🞏1 true 🞏2false  c__________________________________________________🞏1 true 🞏2false |
| 1. List three ways in which animal diseases can be transmitted to humans | a_________________________________________________🞏1 true 🞏2false  b_________________________________________________ 🞏1 true🞏 2false  c_______________________________🞏1 true🞏 2false |
| 1. Name three ways to prevent the transmission of animal diseases to humans | a_________________________________________________🞏1 true🞏 2false  b_________________________________________________ 🞏1 true🞏 2false  c_______________________________🞏1 true🞏 2false |
| Give your opinion to the following statements | |
| 1. All animal diseases can be transmitted to humans | 🞏1 Strongly agree 🞏2 Agree 🞏3 Neither agree nor disagree🞏 4 Disagree 🞏5 Strongly disagree |
| 1. I can get a disease from animals by cleaning their pens | 🞏1 Strongly agree 🞏2 Agree 🞏3 Neither agree nor disagree🞏 4 Disagree 🞏5 Strongly disagree |
| 1. I can contract an animal disease while caring for them | 🞏1 Strongly agree 🞏2 Agree 🞏3 Neither agree nor disagree🞏 4 Disagree 🞏5 Strongly disagree |
| 1. I cannot contract an animal disease while handling a dead animal carcass with my bare hands | 🞏1 Strongly agree 🞏2 Agree 🞏3 Neither agree nor disagree🞏 4 Disagree 🞏5 Strongly disagree |
| 1. I can get an animal disease even if I am not in contact with a sick animal | 🞏1 Strongly agree 🞏2 Agree 🞏3 Neither agree nor disagree🞏 4 Disagree 🞏5 Strongly disagree |
| 1. An apparently healthy animal cannot transmit a disease to humans | 🞏1 Strongly agree 🞏2 Agree 🞏3 Neither agree nor disagree🞏 4 Disagree 🞏5 Strongly disagree |
| **Crimean-Congo hemorrhagic fever** | |
| 1. Have you ever heard of Crimean-Congo Hemorrhagic Fever? | 🞏 Yes 🞏 No |
| 1. If so, what do you call it in your language? |  |
| 1. Is Crimean-Congo hemorrhagic fever an animal disease? | 🞏1 Strongly agree 🞏2 Agree 🞏3 Neither agree nor disagree🞏 4 Disagree 🞏5 Strongly disagree |
| 1. Is Crimean-Congo hemorrhagic fever a disease of humans? | 🞏1 Strongly agree 🞏2 Agree 🞏3 Neither agree nor disagree🞏 4 Disagree 🞏5 Strongly disagree |
| 1. Is Crimean-Congo hemorrhagic fever a zoonosis? | 🞏1 Strongly agree 🞏2 Agree 🞏3 Neither agree nor disagree🞏 4 Disagree 🞏5 Strongly disagree |
| 1. It can be transmitted by tick bites | 🞏1 Strongly agree 🞏2 Agree 🞏3 Neither agree nor disagree🞏 4 Disagree 🞏5 Strongly disagree |
| 1. It can be transmitted by handling animal carcasses when wearing gloves? | 🞏1 Strongly agree 🞏2 Agree 🞏3 Neither agree nor disagree🞏 4 Disagree 🞏5 Strongly disagree |
| 1. It cannot be transmitted by an animal in apparent good health? | 🞏1 Strongly agree 🞏2 Agree 🞏3 Neither agree nor disagree🞏 4 Disagree 🞏5 Strongly disagree |
| 1. An animal suffering from CRIMECONGO fever has serious clinical signs that can lead to death | 🞏1 Strongly agree 🞏2 Agree 🞏3 Neither agree nor disagree🞏 4 Disagree 🞏5 Strongly disagree |
| 1. A human with Crimean-Congo fever may have severe clinical signs that can lead to death | 🞏1 Strongly agree 🞏2 Agree 🞏3 Neither agree nor disagree🞏 4 Disagree 🞏5 Strongly disagree |
| 1. Name three signs of the disease in humans | a_________________________________________________🞏1 true🞏 2false  b_________________________________________________ 🞏1 true🞏 2false  c_______________________________🞏1 true🞏 2false |
| **Attitudes and practices** | |
| 1. In charge of transporting the animals? | 🞏 Yes 🞏 No |
| 1. Clean up the waste in the pens? | 🞏 Yes 🞏 No |
| 1. Gives care to animals (injection, sampling) | 🞏 Yes 🞏 No |
| 1. Milking cows/goats? | 🞏 Yes 🞏 No |
| 1. Washes hands before milking? | 🞏 Yes always 🞏 yes not always 🞏 No |
| 1. Washes hands after milking? | 🞏 Yes always 🞏 yes not always 🞏 No |
| 1. Touch blood (bare hands)? | 🞏 Yes 🞏 No |
| 1. Ever assists in the delivery of animals with your bare hands? | 🞏 Yes 🞏 No |
| 1. Ever slaughter animals with your bare hands? | 🞏 Yes 🞏 No |
| 1. Skinning meat with your bare hands? | 🞏 Yes 🞏 No |
| 1. Touching dead animal carcasses? | 🞏 Yes 🞏 No |
| 1. Average time of contact with animals per day | 🞏Less than one hour 🞏1-6h 🞏6-12H 🞏all day |
| 1. Practical manual tick removal of animals | 🞏 Yes 🞏 No |
| 1. Wear protective equipment during contact with the biological fluid of live or dead animals? | 🞏 Yes always 🞏 yes not always 🞏 No |
| 1. History of tick bites | 🞏 Yes 🞏 No |
| 1. If so, how many times a week on average? | /____/ |
| 1. Would be willing to wear protective equipment before contact with animal secretions? | 🞏 Yes always 🞏 yes not always 🞏 No |
